# Simultaneous Coercivity and Size Determination of Magnetic Nanoparticles

**DOI:** 10.3390/s20143882

**Published:** 2020-07-12

**Authors:** Annelies Coene, Jonathan Leliaert

**Affiliations:** 1 Department of Electromechanical, Systems and Metal Engineering, Ghent University, 9052 Zwijnaarde, Belgium; 2 Cancer Research Institute Ghent, 9000 Ghent, Belgium; 3 Department of Solid State Sciences, Ghent University, 9000 Ghent, Belgium

**Keywords:** magnetorelaxometry, magnetic nanopartices, micromagnetism, nanomagnetism, magnetic dynamics, characterization, size distribution, coercivity, magnetic particle imaging, magnetic particle hyperthermia, 75.78.-n, 75.75.-c, 75.30.Gw, 75.50.Tt, 75.60.Jk

## Abstract

Magnetic nanoparticles are increasingly employed in biomedical applications such as disease detection and tumor treatment. To ensure a safe and efficient operation of these applications, a noninvasive and accurate characterization of the particles is required. In this work, a magnetic characterization technique is presented in which the particles are excited by specific pulsed time-varying magnetic fields. This way, we can selectively excite nanoparticles of a given size so that the resulting measurement gives direct information on the size distribution without the need for any a priori assumptions or complex postprocessing procedures to decompose the measurement signal. This contrasts state-of-the-art magnetic characterization techniques. The possibility to selectively excite certain particle types opens up perspectives in “multicolor” particle imaging, where different particle types need to be imaged independently within one sample. Moreover, the presented methodology allows one to simultaneously determine the size-dependent coercivity of the particles. This is not only a valuable structure–property relation from a fundamental point of view, it is also practically relevant to optimize applications like magnetic particle hyperthermia. We numerically demonstrate that the novel characterization technique can accurately reconstruct several particle size distributions and is able to retrieve the coercivity–size relation of the particles. The developed technique advances current magnetic nanoparticle characterization possibilities and opens up exciting pathways for biomedical applications and particle imaging procedures.

## 1. Introduction

Magnetic nanoparticles (MNPs) exhibit many properties that make them useful for biomedical applications [1,2,3]. For example, their nanoscopic size enables large bioavailability and allows them to interact with cells and viruses. Additionally, the magnetic material of the particles provides opportunities for remote control using external magnetic field gradients, and for noninvasive localization with magnetic sensors. This set of qualities is exploited in numerous biomedical applications [4,5,6]. For instance, in magnetic drug targeting [7], the MNPs act as drug carriers and are guided towards a diseased site where drug release occurs. In disease detection [8], the MNPs attach to the pathogen and reveal its presence and/or location. Finally, in magnetic particle hyperthermia, the MNPs are excited with a strong radio-frequency field in order to locally induce heating to destroy malignant cells [9,10].

An accurate characterization of the nanoparticle properties is important to ensure the reliable, efficient, and safe operation of the applications mentioned above. To this end, several magnetic characterization methods exist in which the MNP response to specific magnetic field sequences is measured to unveil their underlying size distribution [11]; a key parameter in determining application performance [12,13]. In DC magnetometry (DCM) [14], the response to a DC magnetic field is recorded, while in magnetorelaxometry (MRX) [15,16], the MNP’s relaxation is measured after the application of a pulsed DC magnetic field. In AC susceptibility (ACS) [17,18] and Magnetic Particle Spectroscopy (MPS) [19,20], particle properties are retrieved by measuring their response to specific AC fields. Recently, thermal noise magnetometry [21,22] was introduced as a means to overcome possible magnetic field-induced changes to the MNP’s properties (e.g., particle clustering) by measuring the nanoparticles’ magnetic dynamics in the absence of magnetic fields.

These magnetic characterization techniques have in common that they relate the measured MNP dynamics to the particle properties by performing an analysis consisting of the decomposition of the signal into a distribution of size-dependent functions. For example, in MRX the MNP behavior is represented by a superposition of exponentially decaying curves, whose decomposition poses a highly non-trivial mathematical problem [23], and although numerical inversion methods for this problem exist [24,25], the practical feasibility is often improved by introducing assumptions, e.g., that the particles follow a lognormal size distribution [26].

Next to the size distribution, the coercivity of the nanoparticles also has an impact on the application performance. For instance, the heating attained in magnetic nanoparticle hyperthermia strongly depends on the coercivity of the particles [27]. Unfortunately, it is a challenge to accurately determine this quantity because a coercivity measurement is often performed on immobilized particles, while in most applications, the particles are suspended in a liquid. Therefore, the actual coercivity of the particles in the application is not necessarily accurately reflected by the measurements [28]. Furthermore, the measured coercivity value is in fact averaged out over the entire particle size distribution present in the sample. This can be problematic because the coercivity of the MNPs also depends on the nanoparticle size. Therefore, several samples typically need to be prepared for these type of measurements [29,30].

In this paper, an alternative nanoparticle characterization approach is presented that tackles the aforementioned problems: it does not require a complex measurement decomposition with assumptions on particle size distributions and it is able to simultaneously unveil the relation between the coercivity and particle size for suspended particles. This is accomplished by employing specific magnetic fields that exploit the interplay between the magnetic and rotational dynamics of the MNPs, so only particles with a specific coercivity are excited by the applied field. Once the field is removed, a decaying magnetic signal originating from the excited MNPs, similar as in MRX, can be measured using sensitive sensors such as SQUIDs, fluxgates, or optical magnetometers. The main contrast with MRX is that the measured signal only contains the response of particles with a certain coercivity due to the excitation scheme. The particles’ decaying signal then reveals what particle size corresponds to the specific excited coercivity. By changing the properties of the applied magnetic field, the selected particle coercivity, and therefore associated size, can be shifted, resulting in a series of measurements that intrinsically contain particle size information without the need to decompose the resulting measurement data in size-dependent functions in postprocessing.

This paper is organized as follows. The next section presents an overview of the proposed new characterization scheme. This is followed by several sections discussing individual aspects in further detail: first a description is given of the underlying magnetic and rotational dynamics occurring when the MNPs are subjected to magnetic fields (Section 3). In Section 4, different types of AC magnetic fields are investigated for their use in this novel characterization technique. Using the model that was described in Section 3, it is detailed how the MNPs respond to these fields and how this response depends on particle properties like coercivity. This is followed by a discussion on the relation between particle coercivity and size (Section 5). Because we are able to selectively excite particles with a certain coercivity, and thus with a specific size, a single exponentially decaying MNP response at the end of the excitation pulse can be measured. This allows us to determine the particle size related to the excited coercivity, as detailed in Section 6. Section 7 shows the performance of this characterization technique by using it to analyze two distinct MNP systems. This is followed by a discussion on the possible advantages of using our technique in magnetic particle imaging in Section 8, before finishing with the conclusion.

## 2. Overview of the Proposed Characterization Scheme

**Figure 1:**
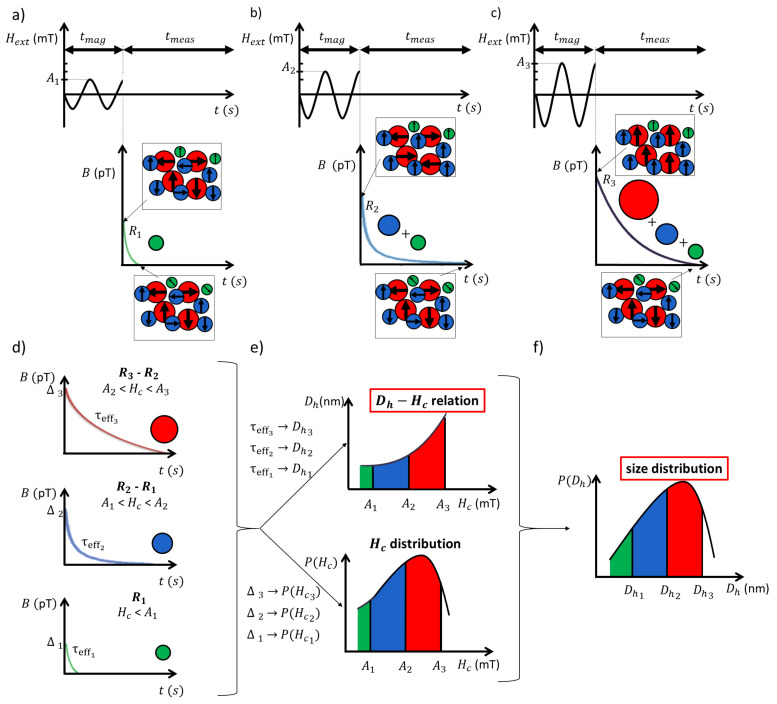
Overview of the different steps of the presented characterization technique for the case of a magnetic nanoparticle (MNP) system consisting of three particle types. In panels (**a**–**c**), the MNP sample is exposed to an AC magnetic field pulse with amplitude $A_{1}$, $A_{2}$, and $A_{3}$, respectively, which is applied for the duration of $t_{\mathrm{mag}}$. Only particles with coercivity lower than the pulse amplitude will align themselves to the pulse direction (inset at beginning of the recording). After the pulse is switched off, this gives rise to a decaying magnetic moment which is recorded for a duration of $t_{\mathrm{mag}}$ seconds. At the end of their relaxation, the particles’ magnetic moments are randomly oriented (inset at the end of the decaying signal). By exposing the sample to pulses of different amplitudes, the associated measurements will contain the response of different sets of particles. In this example, the amplitudes are chosen such that the first pulse (**a**) excites only the first particle type, the second pulse (**b**) the first and second particle type, and the third pulse (**c**) all the particle types. (**d**) Subtracting subsequent measurements allows to retrieve the hydrodynamic diameter ($D_{h}$) and the amount of particles with a specific coercivity ($P(H_{c})$) from the relaxation time and amplitude of the difference signals. (**e**) The information from the difference curves unveils the $D_{h}$ − $H_{c}$ relation and $H_{c}$ distribution for the MNP sample. (**f**) This information can then be combined to retrieve the particle size distribution.

This section illustrates the working principle behind the presented characterization technique by using it on a simplified particle system, containing only three particle types. These particles are distinct from each other in their size *D*, and therefore also have a different coercivity $H_{c}$. In a first step, depicted in Figure 1a, the MNP sample is exposed to an external AC field pulse of duration $t_{\mathrm{mag}}$ with amplitude $A_{1}$. As will be explained in Section 4, only particles with coercive field $H_{c}$ < $A_{1}$ respond to the applied field. These particles are represented in Figure 1a by the green MNPs. As shown in the inset at the end of the field pulse, the magnetic moments of all green MNPs are aligned, resulting in a net magnetic moment originating only from this particle type. When the field is switched off, thermal fluctuations cause the MNPs to slowly reorient their magnetic moments to a random direction (see the inset at the end of the MNP’s response in Figure 1a), causing a relaxation of the net magnetic moment to zero (as will be detailed in Section 6). This decaying signal is captured for $t_{\mathrm{meas}}$ seconds, before a next pulse is applied.

The second pulse, shown in Figure 1b, only varies in amplitude compared to the previously applied pulse. As its amplitude is larger, more particles will meet the requirements to be able to respond to the pulse. In this case, both the green and blue particle types will be excited (see the inset at the end of the pulse). The particles’ response is measured in the same way as was done for the previous pulse, but the decaying signal now contains contributions of both particle types. In Figure 1c, a final field pulse is generated with a sufficient amplitude such that also the last particle type will respond to this pulse. This procedure yields three measurements ($R_{1}$, $R_{2}$, and $R_{3}$) for our simplified example. In practice, a larger number of pulses will be necessary to excite different particles within a sample with a sufficiently fine resolution.

Once the decaying signals are obtained, it is possible to extract the contributions of each individual particle type by subtracting subsequent signals, see Figure 1d. The upper signal is the result of subtracting the measured signal from the third pulse ($R_{3}$) by the one obtained from the second pulse ($R_{2}$). As $R_{3}$ contained contributions of all particle types and $R_{2}$ only contained the response of the blue and green particle types, the specific response of the red particle type is isolated. The middle panel shows the difference between $R_{2}$ and $R_{1}$ and therefore reflects the contributions of the second particle type. The bottom panel depicts the measured signal from the first pulse, which only contained the first particle type. Ideally, the pulse amplitudes are spaced closely, so the difference in decay signals is associated to a small diameter range.

The relaxation times ($\tau_{{\mathrm{eff}}_{1}}$,$\tau_{{\mathrm{eff}}_{2}}$ and $\tau_{{\mathrm{eff}}_{3}}$) and amplitudes ($\Delta_{1}$, $\Delta_{2}$, and $\Delta_{3}$) of these difference curves allow one to extract the particle diameter corresponding to each coercive field and the amount of particles with that specific coercivity ($P(H_{c})$), respectively, as shown in Figure 1e. In this technique the particle size is estimated from the Brownian relaxation time, which only gives information on the hydrodynamic diameter $D_{h}$. This information thus reveals the $D_{h}$ − $H_{c}$ relation and the $H_{c}$ distribution for the sample. By combining this information, the particle size distribution can also be retrieved, as shown in Figure 1f. This example therefore illustrates that our technique simultaneously unveils two critical MNP characteristics: the relation between diameter and coercivity as well as the particle size distribution.

## 3. Magnetic Nanoparticle Dynamics

In the characterization technique presented in this paper, we excite suspended magnetic nanoparticles using magnetic AC fields. To grasp the full complexity of the (interplay between) the magnetic dynamics and Brownian rotation of the particles, it is necessary to numerically integrate the microscopic dynamics.

Let us consider a magnetic nanoparticle with core size $V_{c}$, hydrodynamic volume $V_{h}$, and saturation magnetization $M_{s}$. The magnetization dynamics of this nanoparticle in an externally applied magnetic field is given by the Landau–Lifshitz–Gilbert equation [31].

(1) $$\dot{m}=-\frac{\gamma }{1+\alpha^{2}}\left[m\times H_{\mathrm{eff}}+\alpha m\times \left(m\times H_{\mathrm{eff}}\right)\right]$$

In this equation, m represents the direction of the particle’s magnetization with unit length, and γ and α denote the gyromagnetic ratio and dimensionless Gilbert damping parameter, respectively. It describes the precession around, and damping towards, an effective field $H_{\mathrm{eff}}$, which contains contributions from a (time-dependent) externally applied field $H_{\mathrm{ext}}$ and the (uniaxial) magnetocrystalline anisotropy (with u denoting the direction of the easy axis), whose strength is determined by the anisotropy constant *K*,

(2) $$H_{\mathrm{eff}}=H_{\mathrm{ext}}+\frac{2K}{\mu_{0}M_{\mathrm{sat}}}\left(m\cdot u\right)u+H_{\mathrm{therm}}$$

$\mu_{0}$ denotes the vacuum permeability and $H_{\mathrm{therm}}$ is a stochastic thermal field [32]. The properties of $H_{\mathrm{therm}}$ are such that the thermal fluctuations are uncorrelated in time and space, and that the strength depends on the volume of the particle, leading to physically correct switching times [32].

If, additionally, the particle is suspended in a liquid with viscosity η, its rotational dynamics are described by [33]

(3) $$\dot{u}=\frac{2KV_{c}}{6\eta V_{h}}\left(m\cdot u\right)\left[(m\cdot u)u-m\right]+\theta_{\mathrm{therm}}$$

where the term $\theta_{\mathrm{therm}}$, with similar properties as $H_{\mathrm{therm}}$, accounts for the thermal fluctuations resulting in Brownian (rotational) motion. The rotational dynamics couple to Equations (1) and (2) through the anisotropy terms.

To numerically integrate these equations, we make use of the software package Vinamax [34], which we extended with Equation (3), and which makes use of an efficient algorithm to perform the time-integration of stochastic differential equations [35].

## 4. Magnetic Nanoparticle Response to AC Fields

Current magnetic characterization techniques have in common that all MNP present in the sample simultaneously contribute to the characterization signal. Building upon Usadel et al. in [36], in which sinusoidal varying magnetic fields were employed and a particle-dependent response to these fields was observed, we propose to use these types of fields as a filtering step in the characterization process. This way, a specific subset of the MNP can be targeted, thereby simplifying their characterization. In this section, we investigate the particle’s response to both sinusoidal and rotating externally applied fields and analyze which type of field is most suitable for our purpose. More specifically, we investigate the magnetization of the particle (i.e., the particle’s response) at the end of an applied external field pulse, to see if a discrimination based on particle properties is possible at this time instant. This is important as, in a next step, we want to make use of the magnetic aftereffect to relate particle characteristics to the measured response [37,38] (see Section 6).

### 4.1. Sinusoidal Externally Applied Field

Consider the sinusoidal field described by Equation (4), with amplitude *A* and frequency *f*, applied along the z-direction, $e_{z}$.

(4) $$H_{\mathrm{ext}}=A\sin\left(2\pi ft\right)e_{z}.$$

The resulting response for a given particle can be divided into two dynamical regimes, depending on whether the field amplitude of $H_{\mathrm{ext}}$ is larger or smaller than a critical value [36].

**Figure 2:**
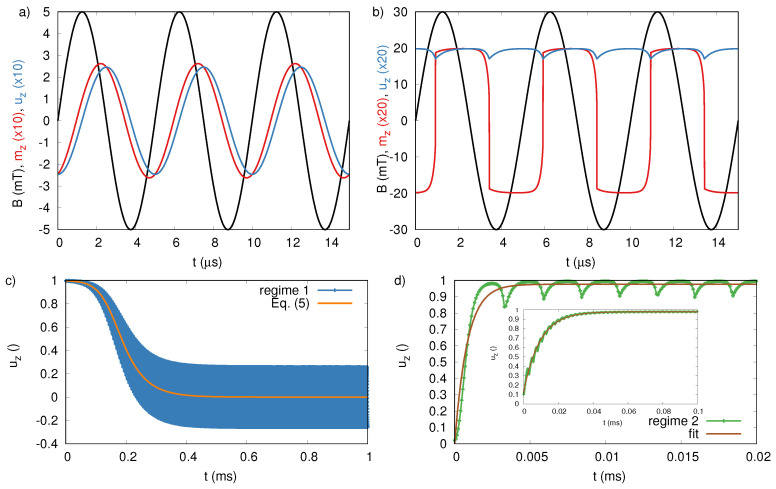
(**a**,**b**) The magnetic field, B, with frequency f=200 kHz, and out-of-plane component of the magnetization, $m_{z}$ , and anisotropy, $u_{z}$, as function of time for a particle with anisotropy field $H_{K}$ = 50 mT. The field amplitude is 5 and 30 mT in panel a and b, respectively, corresponding to the 2 different dynamic regimes in which $u_{z}$ is on average equal to 0 or close to 1. (**c**,**d**) The relaxation of $u_{z}$ towards its dynamical equilibrium value, together with the analytical result of Equation (5) (in regime 1) or the best fit with Equation (7) (in regime 2). Because the dynamic equilibrium is reached very fast for these material parameters, the inset shows a similar result for a particle with $H_{K}$ = 20 mT, excited with a field with amplitude 15 mT, where the validity of the fit (brown line) to Equation (7) is visible over multiple periods of the excitation field.

**Figure 3:**
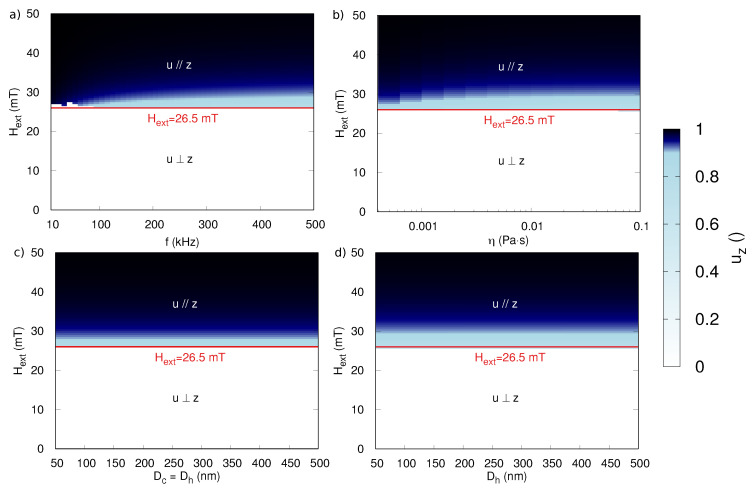
The z-component of the anisotropy, averaged over one period, in dynamical equilibrium during the application of the sinusoidal time-varying field. Panels (**a**,**b**) show $u_{z}$ as function of $H_{\mathrm{ext}}$ for different applied frequencies and viscosities, respectively. Panels (**c**,**d**) show the independence on the size of both the core $D_{c}$ and hydrodynamic $D_{h}$ diameter, both for $D_{c}=D_{h}$ (panel (**c**)) or for varying $D_{h}$ at fixed $D_{c}=$150 nm (panel (**d**)).

Figure 2 illustrates the dynamical equilibrium in both regimes, for an example particle suspended in a liquid with viscosity η = 0.001 Pa·s, corresponding to the viscosity of water at 300 K. In this paper, we will consider thermally blocked magnetic nanoparticles that only show Brownian relaxation. For illustrative purposes, we choose a relatively large particle with size $D_{c}=D_{h}$ = 150 nm and material properties K = 10 kJ/m${}^{3}$ and $M_{\mathrm{sat}}$ = 400 kA/m, similar to iron oxide. This gives rise to an anisotropy field $H_{K}=\frac{2K}{M_{\mathrm{sat}}}$ = 50 mT. The transition of one regime to the other occurs at *A* = 26.5 mT for this particle type, as will be shown below in Figure 3.

In the first regime, of which the dynamic equilibrium is shown in Figure 2a, found for field amplitudes *A* < 26.5 mT, the magnetization follows the direction of the externally applied field with a phase lag. The magnetization dynamics are coupled to the Brownian rotational dynamics through the magnetocrystalline anisotropy (see Equations (1)–(3)). Consequently, the anisotropy axis also follows the magnetization (again with a small phase lag). Interesting in this regime is that the average anisotropy direction lies in the x–y plane ($u⊥e_{z}$). When the applied field is removed, the nanosecond Landau–Lifshitz–Gilbert dynamics will align the magnetization towards the anisotropy easy axes, randomly distributed in this plane, resulting in a zero net magnetization of the particle ensemble. Consequently, in the next step (described in Section 6), no response of the particles will be measured.

This dynamic equilibrium is reached because, during the excitation, the magnetization follows the external field and spends an equal amount of time above and below the x–y plane. Therefore, the anisotropy axis (lagging the magnetization), on average, feels a pull towards this plane.

This regime is not instantaneously reached: the AC field needs to be employed sufficiently long for the anisotropy axis to move towards its dynamic equilibrium value in the x–y plane. We found that these transient dynamics are described by

(5) $$u_{z}\left(t\right)=\frac{\exp\left(-\frac{t}{\tau_{\mathrm{AC}\;1}}-t_{0}\right)}{\sqrt{1+\exp\left(-\frac{2t}{\tau_{\mathrm{AC}\;1}}-2t_{0}\right)}}$$

with $\tau_{\mathrm{AC}\;1}$ a relaxation time constant equal to

(6) $$\tau_{\mathrm{AC}\;1}=\frac{24}{A^{2}}\frac{V_{h}}{V_{c}}\frac{K\eta }{M_{\mathrm{sat}}^{2}},$$

This result can be used to estimate the required application length of the AC field in order to reach the desired dynamic equilibrium state. For the given particle type $\tau_{\mathrm{AC}\;1}$ equals 60 μs.

The validity of this equation is shown in Figure 2c, which shows the motion of u, initialized along the z-axis, towards its equilibrium value (which itself is shown in Figure 2a. The blue line seems quite broad due to the sinusoidal dynamics of u as it follows m and $H_{\mathrm{ext}}$ (clearly visible on the smaller timescale used in panel (a) in the same figure). The average motion of the anisotropy axis towards the horizontal plane almost perfectly follows Equations (5) and (6), as plotted with the orange line.

In the second regime (for *A* > 26.5 mT) of which the dynamical equilibrium is shown in Figure 2b, the magnetic field is sufficiently high to overcome the magnetocrystalline energy barrier and the magnetization makes irreversible jumps from one direction of the anisotropy axis u to the other, as it is pulled towards the positive and negative z-axis by a strong $H_{\mathrm{ext}}$. As a result, the magnetization is almost constantly aligned with the z-axis (either in the positive or negative direction) and forces the anisotropy axis to also align itself with the z-axis ($u\|e_{z}$). This contrasts the first regime, where the dynamical equilibrium direction of the anisotropy axis was in the x–y plane. Consequently, when the applied field is removed at the moment it has maximal amplitude in the (positive) z-direction, the magnetization of all the particles will also be aligned along the (positive) z-axis.

Therefore, in contrast to the first regime, in this regime there is a net magnetization at the end of the excitation pulse allowing a response of the particle to be measured.

Similar as in the first regime, the AC field needs to be applied for a certain time in order to reach the dynamical equilibrium. Figure 2d shows the dynamics of u (green line), as it moves from an initial in-plane position towards its equilibrium direction along the z-axis. This process happens on a faster timescale than the process shown in panel (c). This can be understood from Equation (6) where, $H_{\mathrm{ext}}$, which is larger in this regime, appears quadratically. We found no analytical expression for the average dynamics in this regime, but instead show that it is well described by Equation (7) (see brown line in Figure 2d) .

(7) $$u_{z}\left(t\right)=1-\exp\left(-\frac{t}{\tau_{\mathrm{AC}\;2}}\right)$$

Because the transient state towards dynamic equilibrium is very fast compared to the excitation field frequency for the particle type shown in Figure 2d, the validity of Equation (7) is difficult to assess. Therefore, the inset shows a different particle system with $H_{K}=20$ mT, where the transition happens over a longer timescale, spanning multiple periods of the excitation field, proving that the process can be described by Equation (7). In the inset, the brown line corresponds to a fitted value of $\tau_{\mathrm{AC}\;2}$ = 9.32 μs, which is in reasonable, but not perfect, agreement with the the value estimated from Equation (6), i.e., 10.66 μs. We therefore conclude that Equation (6) can be used as a rough estimate for the timescale on which u reaches its dynamical equilibrium.

Let us now turn our attention to the critical field amplitude at which the transition between both regimes takes place. Figure 3 shows the external field dependence of the average component of the anisotropy along the z-axis, $u_{z}$, as function of different parameters. Unless otherwise stated, the non-varying parameters of the MNP and excitation were the same as in Figure 2: f=200 kHz, $D_{c}$ = 150 nm, $D_{h}=150\;$nm, $H_{K}=50\;$mT, and η=0.001 Pa·s.

As shown in panels (a–d) in Figure 3, the sharp transition between both regimes happens at 26.5 mT, independently of the particle size, the viscosity of the suspension, or the used frequency. This can be understood by realizing that the critical field does not directly depend on these parameters, but rather on the speed at which the different processes take place: as long as the magnetization dynamics happen significantly faster than the Brownian rotation, the direction of *u* does not significantly vary within one period of the external field, and only the ratio between the external field strength and the anisotropy field ($H_{\mathrm{ext}}/H_{K}$) determines whether or not the magnetization can make an irreversible jump to the other magnetocrystalline energy minimum.

**Figure 4:**
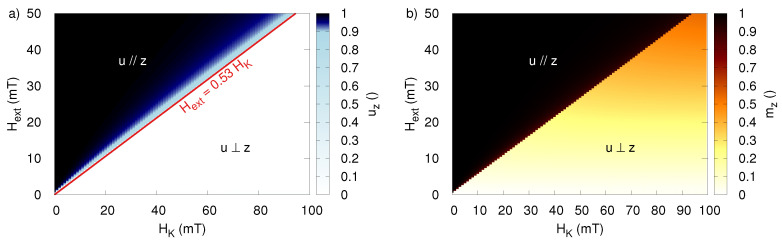
(**a**) The z-component of the anisotropy, averaged over one period, in dynamical equilibrium during the application of the sinusoidal time-varying field as function of the particle coercivity $H_{K}$ and the amplitude of $H_{\mathrm{ext}}$. (**b**) The z-component of the magnetization (i.e., the particles’ response) at the end of the time-varying field pulse.

This picture is further confirmed by Figure 4, which shows a linear relation between the critical field and the anisotropy field of the MNP and corroborates the work in [36], which reports that the sinusoidal applied field for which the transition occurs lies at $0.53H_{K}$. This is also in agreement with the previously found transition at 26.5 mT for particles with $H_{K}$ = 50 mT.

### 4.2. Rotating Externally Applied Field

In the previous discussion of a sinusoidal excitation field, we saw that the magnetization pulled the anisotropy axis either parallel or perpendicular to the z-axis in which the field was applied. The transition between both regimes was determined by whether or not the magnetization showed irreversible switching from a direction close to one side of the anisotropy axis to its opposite side.

Here, we apply a rotating external field, in the plane perpendicular to the z-axis, as described by Equation (8).

(8) $$H_{\mathrm{ext}}=A\sin\left(2\pi ft\right)e_{x}+A\cos\left(2\pi ft\right)e_{y}$$

Extrapolating from the dynamics in a sinusoidal field, we expect that, at low fields, the anisotropy axis will align itself in a direction perpendicular to the applied field. Because the field is applied in the x–y plane, this means $u\|e_{z}$. At high fields, the irreversible switching forces the anisotropy direction to lie in the plane in which the field is applied ($u⊥e_{z}$).

**Figure 5:**
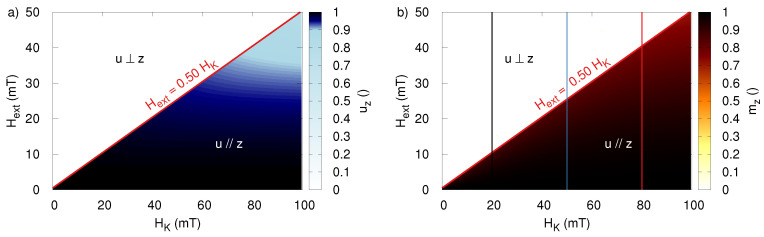
(**a**) The z-component of the anisotropy, averaged over one period, in dynamical equilibrium during the application of the rotating field as function of the particle coercivity $H_{K}$ and the amplitude of $H_{\mathrm{ext}}$. (**b**) The z-component of the magnetization (i.e., the particles’ response) at the end of the time-varying field pulse.

Figure 5a shows the $u_{z}$-component averaged over one period of the external field after it reached its dynamical equilibrium, and confirms that this is indeed the case. The boundary between both dynamical regimes lies at an applied field amplitude of $H_{\mathrm{ext}}$ equal to $0.5H_{K}$. It is interesting to note that this prefactor is slightly different from the prefactor of 0.53 found for a sinusoidal field. Panel (b) shows the magnetization at the end of a rotating magnetic field pulse, and shows that such a pulse can be used to magnetize MNP with an anisotropy field lower than twice the applied external field.

### 4.3. Comparison between Both Excitation Fields

When a set of measurements are performed for varying $H_{\mathrm{ext}}$, a specific subset of particles having a certain $H_{K}$ can be selected by subtracting subsequent measurements. For example, in case of the sinusoidal excitation a measurement for example at 20 mT contains all the particle responses with $H_{K}<10.6$ mT. Likewise, the subsequent measurement at 19 mT will contain slightly fewer particles (only those with $H_{K}<10.07$ mT).

Depending on whether or not $H_{K}$ is inversely proportional to particle size (see Section 5), the signal from the MNP we want to see appears either on top of a larger or smaller background signal, coming from the other excited particles. This means that the signal we are looking for in the differential measurements can originate either from the largest or smallest particles that were excited, making the differential measurements more, or less sensitive.

One advantage of rotating fields is that the details on when and how the excitation field is turned off do not matter as much as in the case of sinusoidal fields, because for sinusoidal fields, the magnetization switches between the positive and negative z-axis during each excitation period.

Furthermore, using rotating fields has the advantage that the particles’ responses in the two regimes are more distinct compared to the sinusoidal excitation (note the sharper transition from white to black for $m_{z}$ in Figure 4b and Figure 5b).

Additionally, because the rotating field is applied in the x–y plane and the net magnetization is perpendicular to this x–y plane, it also allows for sensors to be placed perpendicular to the applied field, facilitating the measurement set-up and signal registration.

Because of these advantages, we will continue to use rotating fields in the remainder of this paper.

## 5. Particle Size-Dependent Coercivity

In the previous section, we showed the feasibility of selectively exciting MNP with a certain coercivity. This section explains how this coercivity is related to the particle size. This link is necessary to be able to connect the measured response of particles to their size and therefore to retrieve the size distribution of the particles in the sample.

The Stoner–Wohlfarth (SW) model predicts no direct size dependence of the nanoparticle coercivity. It states a switching field (necessary to perform an irreversible jump from one anisotropy direction to the other) between 0.5 and 1 times the anisotropy field $H_{K}$, depending on the direction between the anisotropy axis and the applied magnetic field. Due to the ability of suspended magnetic nanoparticles to reorient themselves, the switching field lies close to this minimum value and equals about $0.53H_{K}$ [36] for sinusoidal or $0.5H_{K}$ for rotating fields, as shown above. However, the SW model’s underlying assumption of uniformly magnetized particles with fixed material parameters does not capture the fact that $H_{K}$ itself depends on the particle size.

The size dependence of the coercivity mainly stems from two effects: the size dependence of the material parameters themselves, and the ability of the particle to change magnetization direction via a non-uniform switching process.

The former is mostly relevant for very small MNP with diameters up to several tens of nanometers, and is very complex to describe because the particle surface, which is a crystallographic defect, makes up a large part of the volume. Typically, the surface anisotropy is higher than the bulk anisotropy, causing the coercivity to rise with decreasing size [39]. Moreover, for such small MNP, this effect competes with the Néel relaxation process, in which the magnetization is thermally assisted to jump over the anisotropy energy barrier, causing a reduced nanoparticle coercivity [27,40]. The resulting coercive field for single-domain particles is then given by Equation (9) [41],

(9) $$H_{c}=H_{K}\left[1-{\left(\frac{D_{p}}{D}\right)}^{\frac{3}{2}}\right]$$

where $D_{p}$ is the diameter at which the particles become thermally blocked, i.e., the diameter corresponding to $KV_{p}=25k_{B}T$.

For larger MNP, multidomain structures start to form, allowing the particle to reverse its magnetization through other processes than a uniform rotation, leading to a lower coercivity [42]. For this case, no analytical equation exists, but literature reports an experimentally measured inverse power law relation between the particle size and coercivity, e.g., the authors of [40,41,43] report a $D^{-1}$ dependence of the coercivity, while the work in [30] reports a similar power law, but with an exponent of −0.57±0.06.

We want to emphasize that the method presented in this paper does not depend on the specific relation and also does not require any a priori information on its exact form. In fact, the method presented here allows to determine this relation without making any assumption, while simultaneously determining the particle size distribution. Therefore, we will present results using two very different coercivity–size relations in Section 7.1 and Section 7.2. The latter section also contains a discussion on the reservations related to using our method on multi-domain particles.

## 6. Magnetorelaxometry

Section 4 showed the possibility of exciting nanoparticles with a specific coercivity by applying AC magnetic fields with a certain amplitude. At the end of the field pulse, the magnetic aftereffect can be used to gather information about the particle size distribution [37,38]. This section will detail the impact of this effect on the particles’ measured response and how it can be linked to the particle size distribution. Using this effect has the additional advantage that the relation between $H_{c}$ and particle size can be independently determined, potentially solving conflicting reports in literature. Moreover, by only having a subset of the particles respond to the magnetic field, no a priori assumptions need to be made on the particle distribution.

When the MNP are subjected to a magnetic field that is suddenly removed, the net magnetization of the nanoparticles will not immediately drop to zero, due to a phenomenon called the magnetic aftereffect. Instead, a gradually decaying time signal will be observed in the measurement sensors. This signal is also commonly referred to as the magnetorelaxometry (MRX) signal, as it is observed that the particle ensemble relaxes towards zero net magnetization.

In a typical experiment, this relaxation is measured after the particles are exposed to a static magnetic field to align the magnetization along the field direction. These measurements allow to quantitatively determine, among others, the underlying particle size distribution [38] and the particle location [37,44,45].

The relaxation is driven by two mechanisms that change the orientation of the MNP’s magnetic moment. First, the particle can rotate as a whole, and second, the thermally assisted magnetization dynamics within the core can alter the direction of m. Both mechanisms have an associated time constant called the Brownian relaxation time [46], $\tau_{B}$, and the Néel relaxation time [47,48], $\tau_{N}$, respectively. Both of these processes result from the microscopic dynamics that were described in Section 4 by Equations (1)–(3), and macroscopically give rise to the following size-dependent relaxation time constants

(10) $$\tau_{B}\left(V_{h}\right)=\frac{3\eta V_{h}}{k_{B}T},\;\tau_{N}\left(V_{c}\right)=\tau_{0}\exp\left(\frac{KV_{c}}{k_{B}T}\right)$$

In this equation, $k_{B}$ denotes the Boltzmann constant, *T* the temperature, and $\tau_{0}$ is a time-constant with typical value between $10^{-8}$ s and $10^{-12}$ s. Together they give rise to an effective relaxation time $\tau_{\mathrm{eff}}$ as follows

(11) $$\tau_{\mathrm{eff}}\left(V_{c},V_{h}\right)=\frac{\tau_{N}\left(V_{c}\right)\tau_{B}\left(V_{h}\right)}{\tau_{N}\left(V_{c}\right)+\tau_{B}\left(V_{h}\right)}$$

The relaxing magnetic moment, i.e., the MRX signal, is described by Equation (12).

(12) $$M\left(t\right)=\int_{V_{c}}\int_{V_{h}}M_{0}\left(V_{c},V_{h}\right)\exp\left(\frac{-t}{\tau_{\mathrm{eff}}\left(V_{c},V_{h}\right)}\right)P\left(V_{c},V_{h}\right)dV_{h}dV_{c}$$

Here, $P(V_{c},V_{h})$ denotes the size distribution of the core and hydrodynamic volume of the particles and $M_{0}\left(V_{c},V_{h}\right)$ is the magnetization of the MNP at the end of the magnetic field pulse. In the results section we will consider particles with and without a shell (i.e., $V_{c}$ = $V_{h}$ or $V_{c}$ < $V_{h}$).

The MRX signal can thus be seen as a superposition of different decaying exponential functions with relaxation time constants corresponding to the sizes of the MNP in the sample. Therefore, the particle size distribution can be retrieved by decomposing the MRX signal in its constituent functions. As mentioned in the introduction, however, reconstructing the particle size distribution from the shape of the relaxation curve is not a straightforward problem to solve, and often relies on the assumption of a priori knowledge on the shape of the size distribution. For example, typically, a lognormal size distribution is assumed:

(13) $$P\left(V_{h}\right)=\frac{1}{\sqrt{2\pi }\sigma_{h}V_{h}}\exp\left(-\frac{\ln^{2}\left(V_{h}/\mu_{h}\right)}{2\sigma_{h}^{2}}\right)$$

where $\mu_{h}$ is the median particle size and $\sigma_{h}$ its geometric standard deviation. By fitting Equation (12) with parameters $\mu_{h}$ and $\sigma_{h}$ to a measured MRX curve, the particle size distribution can be determined. Nevertheless, the lognormal distribution is not always a correct assumption [49]. Recently, an approach was presented which does not rely on such assumptions, and instead uses the iterative Kaczmarz’ algorithm to decompose the signal in its constituent functions, as demonstrated for both DCM [50] and MRX [51]. However, due to the self-similar nature of these curves, this approach also has its limitations, and especially has difficulties with non-smooth size distributions displaying features like sharp peaks.

In this paper, we will adopt a direct measurement scheme in which the previous decomposition is not longer necessary. Instead, time-varying fields will be applied during the excitation phase in order to only excite a subsection of the particles (see Section 4). By performing a series of such (simulated) measurements, and subtracting subsequent results, only a specific particle size will be present in each decaying signal. Therefore, it is possible to directly determine the size distribution of the particles, as the decaying signal will be a single exponential. Additionally, this approach allows to determine the particle’s coercive field as function of its size.

## 7. Characterization Results

We now come to the key result of our manuscript, i.e., how to simultaneously determine the MNP size and coercivity. To this end, a series of (simulated) measurements are performed in which the sample is excited with a rotating magnetic field, and afterwards the resulting relaxation is measured. By repeating this measurement for increasing field amplitudes, we can selectively excite particles with a specific coercivity range. By subtracting subsequent measurement signals we can (1) deduce the amount of MNP with that specific coercivity from the amplitude of the signal and (2) deduce the size of these particles from the relaxation time constant. This procedure thus yields simultaneous information on the size-dependent coercivity of the particles and on the particle size distribution.

**Figure 6:**
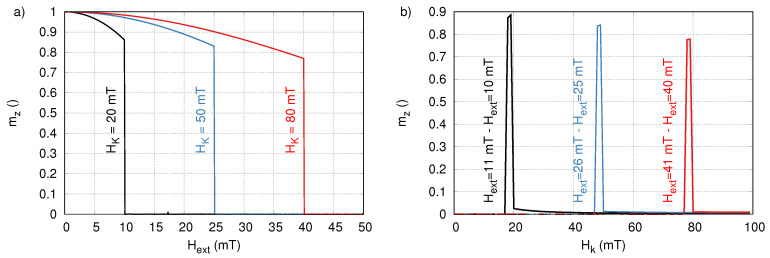
(**a**) The z-component of the magnetization as function of externally applied rotating field amplitude for particles with 3 different anisotropy fields, corresponding to 3 cross sections of Figure 5b (indicated with lines in the same color). (**b**) Three examples of the difference between two subsequent excitations with pulse amplitudes spaced 1 mT apart. This shows that it is possible to excite a very narrow subset of the particles with a specific $H_{K}$, and thus a specific coercivive field of $H_{K}/2$.

Figure 6 shows the calculated magnetization (see Section 4) as function of the amplitude of $H_{\mathrm{ext}}$ for three different $H_{K}$, corresponding to the three vertical lines in Figure 5b. The figure shows that the magnetization performs a large jump at $H_{\mathrm{ext}}=H_{c}=0.5H_{K}$. However, the magnetization does not jump all the way from $m_{z}=0$ to $m_{z}=1$, because it oscillates around its average value as it follows $H_{\mathrm{ext}}$. This effect can be reduced, e.g., by letting $H_{\mathrm{ext}}$ die out exponentially, or by increasing the field frequency (or sample viscosity) to allow less time for the particle to follow the excitation field. Here, we present a worst case scenario, in which this effect is larger than it would be in most measurements, and it is further amplified by the fact that we consider a large $H_{\mathrm{ext}}$ amplitude range from 0 to 50 mT.

Panel (b) in Figure 6 shows three examples of the difference between two subsequent excitations (spaced 1 mT apart) and proves that it is possible to very selectively excite part of the sample with a specific coercive field. Note that in principle these peaks can be made even narrower by reducing the amplitude difference in subsequent excitation fields, albeit at the cost of additional measurements.

We now turn our attention to the performance of the presented method by extracting the coercivity and size distribution of two distinct MNP samples.

### 7.1. Sample 1: Lognormal Distribution of Single-Domain Particles

The first simulated sample consists of magnetic nanoparticles with material properties similar to those of iron oxide [52] (i.e., K=10 kJ/m${}^{3}$, $A_{\mathrm{ex}}=20$ pJ/m, and $M_{\mathrm{sat}}=400$ kA/m). We assume a lognormal core size distribution with $\mu_{c}$ = 50 nm and $\sigma_{c}$ = 0.15, surrounded by a 10 nm shell. Furthermore, we use T=290 K and η=40 mPA s, corresponding to a 80% glycerol solution at room temperature.

These parameters yield an anisotropy field $H_{K}$ of 50 mT, $D_{p}=26$ nm, above which the particles are thermally blocked and a diameter $D_{c}=102$ nm, below which the particles have a single-domain magnetization [53]. The size distribution of the particles was chosen to fall well within these bounds. The coercive field of the particles is thus given by Equation (9) as

(14) $$H_{c}=50\mathrm{mT}\left[1-{\left(\frac{27\mathrm{nm}}{D_{c}}\right)}^{\frac{3}{2}}\right]$$

The MRX measurements were simulated as follows. The total initial amplitude of the MRX signal is the integral over of the magnetization expected at the end of rotating magnetic field pulses with a frequency of 200 kHz and amplitudes ranging from 1 to 50 mT, weighted with the particle moment ($M_{\mathrm{sat}}V_{c}$) and the particle size distribution P($V_{c}$).

(15) $$M_{0}=\int_{V_{c}}M_{0}\left(V_{c}\right)P\left(V_{c}\right)dV_{c}=\int_{V_{c}}M_{\mathrm{sat}}V_{c}m_{z}\left(V_{c}\right)P\left(V_{c}\right)dV_{c}$$

The corresponding relaxation curves are then generated using Equation (12), and subsequent measurements (corresponding to different amplitudes of the excitation field) are subtracted from each other. For perfectly narrow peaks, this would lead to an exponentially decaying curve with relaxation time constant corresponding to a single particle size having $H_{c}$ equal to the amplitude of $H_{\mathrm{ext}}$.

**Figure 7:**
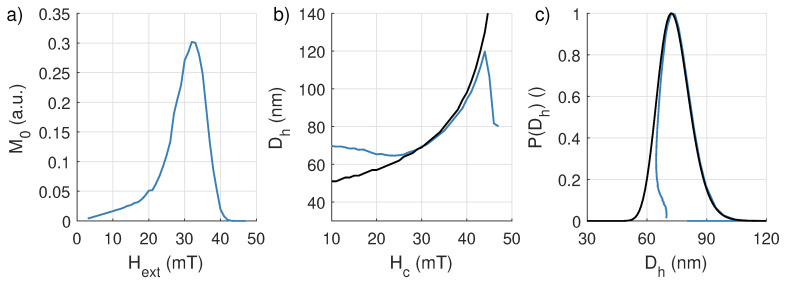
(**a**) Initial particle response ($M_{0}$) at the end of the excitation. (**b**) Extracted $D_{h}$ by fitting an exponentially decaying curve to each MRX measurement. The blue line corresponds to the extracted particle size of the MRX measurements, and the black line is the actual coercivity. (**c**) The reconstructed size distribution (blue line) and the actual particle size distribution (black line).

The initial amplitude of this differential curve is a direct measure for $P(H_{c})$, as shown in panel (a) of Figure 7. In the following two steps of our analysis, we will (1) determine the relation between $H_{c}$ and $D_{h}$, and (2) use this relation to transform $P(H_{c})$ into $P(D_{h})$.

(1) An exponentially decaying function is fitted to each measurement to retrieve the associated particle size for each coercivity, shown in Figure 7b. The black line represents the actual $H_{c}$—size relation (Equation (14)) and the blue line is the extracted relation by fitting the exponential functions. As can be observed, for lower $H_{c}$-values it becomes harder to find an accurate exponential fit, and the estimated diameter $D_{h}$ corresponding to these lower $H_{c}$ strongly deviates from the black line before settling at a lower value. This can be understood from Figure 6a. Indeed, there it is shown that the magnetization response does not jump exactly from 0 to 1, but to a value slightly lower than 1, which is different for each $H_{c}$. Therefore, when two measurements are subtracted there is a small but non-zero signal left for $H_{c}$ > $H_{\mathrm{ext}}$ . This is especially relevant for lower applied external fields and thus smaller coercivities (see the tail to the right of the black peak in Figure 6b). In this case, our differential measurement does not only contain the signal we expect, but also contains a long tail of signals originating from MNP in the distribution with $H_{c}$ > $H_{\mathrm{ext}}$. An improved transition can be realized by further optimizing external field parameters to minimize the deviation from a perfect jump of the magnetization from 0 to 1, e.g., by employing a larger excitation frequency. Furthermore, at large $H_{c}$ > 45 mT, a deviation from the black line is observed, which is attributed to a lack of measurement signal, as can be seen in panel (a).

(2) We can now use the obtained relation between $H_{c}$ and $D_{h}$ to transform $P(H_{c})$ into $P(D_{h})$. The results of this procedure are shown in panel (c) of Figure 7). The black line corresponds to the actual size distributions, while the blue line shows the distributions obtained directly from the simulated measurements and the exponential fits.

### 7.2. Sample 2: Bimodal Distribution of Multi-Domain Particles

For the second sample, we consider larger particles with a bimodal distribution generated by taking the sum of two lognormal distributions having parameters $\mu_{h}$= 90 nm and 150 nm and both having a $\sigma_{h}$ = 0.1.

The applicability of our method to particles in a multi-domain state, which is the expected state for these sizes, is not evident. The previous sections were based on the assumption that the dynamics can be described by a single macrospin, governed by Equations (1) and (2). More specifically, this precludes the possibility for the particles to switch magnetization state from one (local) minimum to another without overcoming the anisotropy field $H_{K}$ (multiplied by a factor 0.5 or 0.53 when accounting for the rotational dynamics described by Equation (3)). This is, however, not central to our characterization method per se: the fact that coercive fields are still experimentally observed for such particles means that there is still an energy barrier that needs to be overcome to switch the magnetization direction. This energy barrier will be lower than what it would have been for a single-domain particle switching via uniform rotation, but the fact remains that a coercive field $H_{c}$ is required to overcome it.

The physical mechanism that dictated the response to AC fields (detailed in Section 4) was whether or not the applied external field was sufficient to overcome the anisotropy energy barriers and allow the magnetization to switch directions to the opposite energy minimum. Given that this is equally applicable to particles in a multi-domain state, makes it plausible that our method can also be used for such particles.

Therefore, we continue by following literature (see Section 5) and assume an inverse relation between the particle size and its coercive field (Equation (16)) and repeat the characterization procedure described for sample 1.

(16) $$D_{h}=\frac{2000\;\mathrm{mT}\cdot \mathrm{nm}}{H_{c}}$$

**Figure 8:**
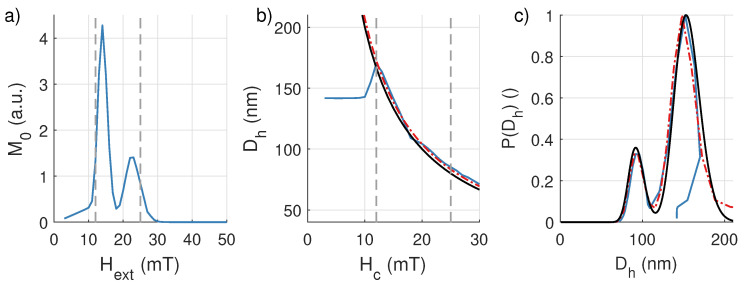
(**a**) Initial particle response ($M_{0}$) at the end of the excitation. The dashed lines show the subset of measurements for which the signal is larger than 25% of its maximum value. (**b**) Extracted $D_{h}$ by fitting an exponentially decaying curve to each MRX measurement. The blue line corresponds to the extracted particle size of the MRX measurements, the black line is the actual coercivity–size relation and the red dash-dotted line represents the fitted coercivity–size relation based on the measurement values (in blue) between the two dashed lines. (**c**) The black line represents the actual particle size distribution, the blue line is the extracted size distribution based on all the measurements, and the red dash-dotted line is the found distribution using the fitted relation between size–coercivity for only part of the measurement data.

The results of this procedure are shown in Figure 8.

Panel (a) again shows the initial magnetization obtained from the difference between relaxation curves resulting from consecutive excitations, while panel (b) shows the hydrodynamic size extracted from the relaxation curves as function of the coercive field. When using the result shown in panel (b) to transform the result form panel (a) into a size distribution, we get the blue line shown in panel (c).

Note that panel (b) shows very similar behavior as the corresponding panel in Figure 7, i.e., at low coercive fields, the line drops off from the black line corresponding to the real diameter. However, because of the inverse relation between coercivity and size, this now gives rise to an inaccurate reconstructed diameter distribution for *large* diameters, as shown in panel (c). This shows that, in this regime, our method can also be used to complement other methods, in which the smallest particles are typically the hardest to accurately reconstruct.

One way to mitigate the inaccuracies for small coercive fields, and improve the performance of our technique, is to only consider the region where the obtained signals are larger than 25% of the maximal amplitude (i.e., the region between the gray dashed lines). Within this region, the size–coercivity relation can then be fitted to an inverse power law of the form

(17) $$D_{h}=a_{1}H_{c}^{-a_{2}}$$

with fit parameters {$a_{1}$, $a_{2}$}. For the sample under study, we found $a_{1}$ and $a_{2}$ to be equal to 2036 and 0.99, respectively (red line, in panel (b) of Figure 8). These values are in close agreement with Equation (16), proving that the presented method can be used to accurately determine the size–coercivity relation, if sufficient signal is measured.

When using the fitted relation between $H_{c}$ and $D_{h}$ to retrieve the size distribution of the particles (red dash-dotted line in panel (c) in Figure 8), we find that also an accurate characterization can be performed over the entire size distribution.

We summarize the results section by repeating that Figure 7 and Figure 8 show that the presented technique can accurately determine the coercivity–size relation of the MNP and simultaneously allows one to retrieve their size distribution (both of a lognormal and bimodal distribution) without the need for any assumptions or complex signal decompositions in postprocessing. We have presented an argument that, next to single-domain particles, our method plausibly is also applicable to multi-domain particles, although further study will be necessary to verify this.

## 8. Potential Use in Magnetic Nanoparticle Imaging

The presented method also has potential in “multi-color” or “multiplexed” [54] imaging, i.e., the simultaneous imaging of multiple nanoparticle types. This is especially useful in a theranostic [55] approach, where different particle types, optimized for a different function (disease detection, drug delivery, hyperthermia, etc.) are used simultaneously. One prerequisite for such applications to work is the possibility to separately image the spatial distribution of each of these particle types. The feasibility of such imaging has been demonstrated already both in MPI (for three different particle types) [56] and MRX [44] (for four different particle types), by decomposing a convoluted signal in postprocessing. Our technique would allow to significantly increase the number of different MNP types that can be imaged. This is because the applied AC fields can selectively excite specific MNP types in the sample, compared to previous approaches that excite all particle types simultaneously by applying DC fields instead [44,57]. Additionally, our technique eliminates the need for heavy postprocessing as no different particle types in the measurement signal need to be discerned.

In a biomedical setting, it is possible that all MNP types have a similar size, as they are optimized towards biocompatibility (e.g., uptake/recognition by specific biological entities). Provided that the different particle types have a distinct coercivity (e.g., due to a varying chemical composition of the core), our approach would still be able to discern the particle types, whereas multi-color MRX would not see any difference as the relaxometry signals of the MNP types would be identical. Therefore, our technique could be very relevant in biomedical settings.

Furthermore, by combining different excitation fields (sinusoidal, or rotating fields, as presented in Section 4), it is feasible to excite either particles with a low, or high coercivity, respectively, allowing to increase the particle information content in the MRX signal, therefore improving imaging performance [58]. Moreover, in line with current progress in MRX imaging, in which it is investigated what magnetic fields need to be applied to improve the quality of the MRX images [59], the AC fields could be combined with the currently existing DC approaches to tackle this problem.

A detailed study of the performance of such an imaging approach lies beyond the scope of this work, and will be presented separately in future work.

## 9. Conclusions

In this paper, we present a magnetic nanoparticle characterization technique in which AC magnetic field pulses are applied to the particles, with the aim to selectively excite particles with a specific coercivity. Literature shows that the coercivity is linked to the particle size, and therefore, this technique can be used for an accurate size distribution determination. To determine the relation between nanoparticle coercivity and size, we measure the time-decaying signal of the particles at the end of the excitation field pulse, as it contains information on the particle size. In our case, the signal consists of only one single exponentially decaying curve with a time constant corresponding to the nanoparticle size. This allows one to significantly simplify the characterization of the particles as no complex decomposition of the signal is necessary and no a priori assumptions on particle distribution need to be made, as is required for other magnetic characterization techniques. The performance of this technique was demonstrated by accurately characterizing a lognormal sample distribution and a biomodal one, additionally proving the versatility of the presented approach.

A second advantage of the technique is that it uniquely provides information on the size–coercivity relation of the MNP without averaging over all diameters present in the sample. It can therefore be used to investigate this structure–property relation without the need to prepare multiple monodisperse samples, as is usually the case in literature. Our technique might therefore help to shed light onto the the different inverse power laws reported in literature.

Knowledge on the size-coercivity relation is not only important from a fundamental point of view. It is also crucial for magnetic hyperthermia applications, because the nanoparticle heating is strongly dependent on the coercivity of the particles. Using the presented method to characterize the particles therefore allows one to optimize magnetic field parameters towards the sweet spot of maximal particle heating with minimal energy consumption.

Finally, the presented technique has potential use in “multicolor” or “multiplexed” imaging in which the spatial location of multiple particle types needs to be determined simultaneously. Our technique could significantly improve the imaging quality by selectively exciting different particle types (provided that they have different coercivities), even when all of them have the same size optimized towards biocompatibility.

In conclusion, a new characterization technique is presented that allows one to simultaneously determine the size–coercivity relation of magnetic nanoparticles as well as to accurately retrieve their size distribution. It contrasts other magnetic characterization techniques by the fact that no assumptions need to be made on the shape of the particle size distribution, nor does it involve a complex decomposition of superimposed signals. It therefore advances general characterization beyond the state-of-the-art and allows to improve the efficiency and safety of MNP-based applications.

## References

[1] Pankhurst Q., Connolly J., Jones S., Dobson J. (2003). Applications of magnetic nanoparticles in biomedicine. J. Phys. D Appl. Phys..

[2] Pankhurst Q.A., Thanh N.T.K., Jones S.K., Dobson J. (2009). Progress in applications of magnetic nanoparticles in biomedicine. J. Phys. D Appl. Phys..

[3] Wu W., Wu Z., Yu T., Jiang C., Kim W.S. (2015). Recent progress on magnetic iron oxide nanoparticles: Synthesis, surface functional strategies and biomedical applications. Sci. Technol. Adv. Mater..

[4] Dadfar S.M., Roemhild K., Drude N.I., von Stillfried S., Knüchel R., Kiessling F., Lammers T. (2019). Iron oxide nanoparticles: Diagnostic, therapeutic and theranostic applications. Adv. Drug Deliv. Rev..

[5] Tong S., Zhu H., Bao G. (2019). Magnetic iron oxide nanoparticles for disease detection and therapy. Mater. Today.

[6] Reddy L.H., Arias J.L., Nicolas J., Couvreur P. (2012). Magnetic nanoparticles: Design and characterization, toxicity and biocompatibility, pharmaceutical and biomedical applications. Chem. Rev..

[7] Al-Jamal K.T., Bai J., Wang J.T.W., Protti A., Southern P., Bogart L., Heidari H., Li X., Cakebread A., Asker D. (2016). Magnetic Drug Targeting: Preclinical in Vivo Studies, Mathematical Modeling, and Extrapolation to Humans. Nano Lett..

[8] De Haro L.P., Karaulanov T., Vreeland E.C., Anderson B., Hathaway H.J., Huber D.L., Matlashov A.N., Nettles C.P., Price A.D., Monson T.C. (2015). Magnetic relaxometry as applied to sensitive cancer detection and localization. Biomed. Eng. Tech..

[9] Dennis C.L., Ivkov R. (2013). Physics of heat generation using magnetic nanoparticles for hyperthermia. Int. J. Hyperth..

[10] Périgo E.A., Hemery G., Sandre O., Ortega D., Garaio E., Plazaola F., Teran F.J. (2015). Fundamentals and advances in magnetic hyperthermia. Appl. Phys. Rev..

[11] Bogren S., Fornara A., Ludwig F., del Puerto Morales M., Steinhoff U., Hansen M.F., Kazakova O., Johansson C. (2015). Classification of Magnetic Nanoparticle Systems—Synthesis, Standardization and Analysis Methods in the NanoMag Project. Int. J. Mol. Sci..

[12] Harabech M., Leliaert J., Coene A., Crevecoeur G., Van Roost D., Dupré L. (2017). The effect of the magnetic nanoparticle’s size dependence of the relaxation time constant on the specific loss power of magnetic nanoparticle hyperthermia. J. Magn. Magn. Mater..

[13] Ferguson R.M., Khandhar A.P., Kemp S.J., Arami H., Saritas E.U., Croft L.R., Konkle J., Goodwill P.W., Halkola A., Rahmer J. (2015). Magnetic Particle Imaging With Tailored Iron Oxide Nanoparticle Tracers. IEEE Trans. Med. Imaging.

[14] Van Rijssel J., Kuipers B.W., Erné B.H. (2014). Non-regularized inversion method from light scattering applied to ferrofluid magnetization curves for magnetic size distribution analysis. J. Magn. Magn. Mater..

[15] Ludwig F., Mäuselein S., Heim E., Schilling M. (2005). Magnetorelaxometry of magnetic nanoparticles in magnetically unshielded environment utilizing a differential fluxgate arrangement. Rev. Sci. Instrum..

[16] Lange J., Kotitz R., Haller A., Trahms L., Semmler W., Weitschies W. (2002). Magnetorelaxometry—A new binding specific detection method based on magnetic nanoparticles. J. Magn. Magn. Mater..

[17] Ludwig F., Balceris C., Jonasson C., Johansson C. (2017). Analysis of ac susceptibility spectra for the characterization of magnetic nanoparticles. IEEE Trans. Magn..

[18] Dieckhoff J., Eberbeck D., Schilling M., Ludwig F. (2016). Magnetic-field dependence of Brownian and Néel relaxation times. J. Appl. Phys..

[19] Wawrzik T., Schilling M., Ludwig F. (2012). Perspectives of magnetic particle spectroscopy for magnetic nanoparticle characterization. Magnetic Particle Imaging.

[20] Draack S., Lucht N., Remmer H., Martens M., Fischer B., Schilling M., Ludwig F., Viereck T. (2019). Multiparametric magnetic particle spectroscopy of CoFe2O4 nanoparticles in viscous media. J. Phys. Chem. C.

[21] Leliaert J., Coene A., Liebl M., Eberbeck D., Steinhoff U., Wiekhorst F., Fischer B., Dupré L., Van Waeyenberge B. (2015). Thermal magnetic noise spectra of nanoparticle ensembles. Appl. Phys. Lett..

[22] Leliaert J., Eberbeck D., Liebl M., Coene A., Steinhoff U., Wiekhorst F., Van Waeyenberge B., Dupré L. (2017). The complementarity and similarity of magnetorelaxometry and thermal magnetic noise spectroscopy for magnetic nanoparticle characterization. J. Phys. D Appl. Phys..

[23] Istratov A.A., Vyvenko O.F. (1999). Exponential analysis in physical phenomena. Rev. Sci. Instrum..

[24] Bender P., Balceris C., Ludwig F., Posth O., Bogart L., Szczerba W., Castro A., Nilsson L., Costo R., Gavilán H. (2017). Distribution functions of magnetic nanoparticles determined by a numerical inversion method. New J. Phys..

[25] Bender P., Fock J., Frandsen C., Hansen M.F., Balceris C., Ludwig F., Posth O., Wetterskog E., Bogart L.K., Southern P. (2018). Relating magnetic properties and high hyperthermia performance of iron oxide nanoflowers. J. Phys. Chem. C.

[26] Eberbeck D., Wiekhorst F., Steinhoff U., Trahms L. (2006). Aggregation behaviour of magnetic nanoparticle suspensions investigated by magnetorelaxometry. J. Phys. Condens. Matter.

[27] Starsich F.H.L., Eberhardt C., Boss A., Hirt A.M., Pratsinis S.E. (2018). Coercivity Determines Magnetic Particle Heating. Adv. Healthc. Mater..

[28] Hergt R., Hiergeist R., Zeisberger M., Glöckl G., Weitschies W., Ramirez L., Hilger I., Kaiser W.A. (2004). Enhancement of AC-losses of magnetic nanoparticles for heating applications. J. Magn. Magn. Mater..

[29] Hergt R., Dutz S., Röder M. (2008). Effects of size distribution on hysteresis losses of magnetic nanoparticles for hyperthermia. J. Phys. Condens. Matter..

[30] Heider F., Dunlop D.J., Sugiura N. (1987). Magnetic Properties of Hydrothermally Recrystallized Magnetite Crystals. Science.

[31] Landau L., Lifshitz E. (1935). Theory of the dispersion of magnetic permeability in ferromagnetic bodies. Phys. Z. Sowietunion.

[32] Brown W.F. (1963). Thermal Fluctuations of a Single-Domain Particle. Phys. Rev..

[33] Reeves D.B., Weaver J.B. (2015). Combined Néel and Brown rotational Langevin dynamics in magnetic particle imaging, sensing, and therapy. Appl. Phys. Lett..

[34] Leliaert J., Vansteenkiste A., Coene A., Dupré L., Van Waeyenberge B. (2015). Vinamax: A macrospin simulation tool for magnetic nanoparticles. Med. Biol. Eng. Comput..

[35] Leliaert J., Mulkers J., De Clercq J., Coene A., Dvornik M., Van Waeyenberge B. (2017). Adaptively time stepping the stochastic Landau-Lifshitz-Gilbert equation at nonzero temperature: Implementation and validation in MuMax3. AIP Adv..

[36] Usadel K.D., Usadel C. (2015). Dynamics of magnetic single domain particles embedded in a viscous liquid. J. Appl. Phys..

[37] Liebl M., Wiekhorst F., Eberbeck D., Radon P., Gutkelch D., Baumgarten D., Steinhoff U., Trahms L. (2015). Magnetorelaxometry procedures for quantitative imaging and characterization of magnetic nanoparticles in biomedical applications. Biomed. Eng. Tech..

[38] Wiekhorst F., Steinhoff U., Eberbeck D., Trahms L. (2012). Magnetorelaxometry assisting biomedical applications of magnetic nanoparticles. Pharm. Res..

[39] Berger L., Labaye Y., Tamine M., Coey J.M.D. (2008). Ferromagnetic nanoparticles with strong surface anisotropy: Spin structures and magnetization processes. Phys. Rev. B.

[40] Luna C., del Puerto Morales M., Serna C.J., Vázquez M. (2003). Multidomain to single-domain transition for uniform Co80Ni20nanoparticles. Nanotechnology.

[41] Kneller E.F., Luborsky F.E. (1963). Particle Size Dependence of Coercivity and Remanence of Single-Domain Particles. J. Appl. Phys..

[42] Brown W.F. (1969). The fundamental theorem of the theory of fine ferromagnetic particles. Ann. New York Acad. Sci..

[43] Lee J.S., Cha J.M., Yoon H.Y., Lee J.K., Kim Y.K. (2015). Magnetic multi-granule nanoclusters: A model system that exhibits universal size effect of magnetic coercivity. Sci. Rep..

[44] Coene A., Leliaert J., Liebl M., Löwa N., Steinhoff U., Crevecoeur G., Dupré L., Wiekhorst F. (2017). Multi-color magnetic nanoparticle imaging using magnetorelaxometry. Phys. Med. Biol..

[45] Jaufenthaler A., Schier P., Middelmann T., Liebl M., Wiekhorst F., Baumgarten D. (2020). Quantitative 2D magnetorelaxometry imaging of magnetic nanoparticles using optically pumped magnetometers. Sensors.

[46] Einstein A. (1956). Investigations on the Theory of the Brownian Movement.

[47] Louis N. (1950). Théorie du traînage magnétique des substances massives dans le domaine de Rayleigh. J. Phys. Radium.

[48] Leliaert J., Coene A., Crevecoeur G., Vansteenkiste A., Eberbeck D., Wiekhorst F., Van Waeyenberge B., Dupré L. (2014). Regarding the Néel relaxation time constant in magnetorelaxometry. J. Appl. Phys..

[49] Eberbeck D., Wiekhorst F., Wagner S., Trahms L. (2011). How the size distribution of magnetic nanoparticles determines their magnetic particle imaging performance. Appl. Phys. Lett..

[50] Schmidt D., Eberbeck D., Steinhoff U., Wiekhorst F. (2016). Finding the magnetic size distribution of magnetic nanoparticles from magnetization measurements via the iterative Kaczmarz algorithm. J. Magn. Magn. Mater..

[51] Leliaert J., Schmidt D., Posth O., Liebl M., Eberbeck D., Coene A., Steinhoff U., Wiekhorst F., Waeyenberge B.V., Dupré L. (2017). Interpreting the magnetorelaxometry signal of suspended magnetic nanoparticles with Kaczmarz’ algorithm. J. Phys. D Appl. Phys..

[52] Usov N.A. (2019). Iron Oxide Nanoparticles for Magnetic Hyperthermia. SPIN.

[53] Fratta G.D., Serpico C., d’Aquino M. (2012). A generalization of the fundamental theorem of Brown for fine ferromagnetic particles. Phys. B Condens. Matter.

[54] Fan Y., Wang P., Lu Y., Wang R., Zhou L., Zheng X., Li X., Piper J.A., Zhang F. (2018). Lifetime-engineered NIR-II nanoparticles unlock multiplexed in vivo imaging. Nat. Nanotechnol..

[55] Zhu L., Zhou Z., Mao H., Yang L. (2017). Magnetic nanoparticles for precision oncology: Theranostic magnetic iron oxide nanoparticles for image-guided and targeted cancer therapy. Nanomedicine.

[56] Rahmer J., Halkola A., Gleich B., Schmale I., Borgert J. (2015). First experimental evidence of the feasibility of multi-color magnetic particle imaging. Phys. Med. Biol..

[57] Coene A., Crevecoeur G., Dupré L. (2012). Adaptive control of excitation coil arrays for targeted magnetic nanoparticle reconstruction using magnetorelaxometry. IEEE Trans. Magn..

[58] Coene A., Leliaert J., Dupré L., Crevecoeur G. (2015). Quantitative model selection for enhanced magnetic nanoparticle imaging in magnetorelaxometry. Med. Phys..

[59] Schier P., Liebl M., Steinhoff U., Handler M., Wiekhorst F., Baumgarten D. (2020). Optimizing excitation coil currents for advanced magnetorelaxometry imaging. J. Math. Imaging Vis..

